# Diverse Immunological Factors Influencing Pathogenesis in Patients with COVID-19: A Review on Viral Dissemination, Immunotherapeutic Options to Counter Cytokine Storm and Inflammatory Responses

**DOI:** 10.3390/pathogens10050565

**Published:** 2021-05-07

**Authors:** Ali A. Rabaan, Shamsah H. Al-Ahmed, Mohammed A. Garout, Ayman M. Al-Qaaneh, Anupam A Sule, Raghavendra Tirupathi, Abbas Al Mutair, Saad Alhumaid, Abdulkarim Hasan, Manish Dhawan, Ruchi Tiwari, Khan Sharun, Ranjan K. Mohapatra, Saikat Mitra, Talha Bin Emran, Muhammad Bilal, Rajendra Singh, Salem A. Alyami, Mohammad Ali Moni, Kuldeep Dhama

**Affiliations:** 1 Molecular Diagnostic Laboratory, Johns Hopkins Aramco Healthcare, Dhahran 31311, Saudi Arabia; ali.rabaan@jhah.com; 2 Specialty Paediatric Medicine, Qatif Central Hospital, Qatif 32654, Saudi Arabia; shalahmed@moh.gov.sa; 3 Department of Community Medicine and Health Care for Pilgrims, Faculty of Medicine, Umm Al-Qura University, Makkah 21955, Saudi Arabia; magarout@sghgroup.net; 4 Department of Genetic Research, Institute for Research and Medical Consultations (IRMC), Imam Abdulrahman Bin Faisal University, Dammam 31441, Saudi Arabia; ayman.qaaneh@jhah.com; 5 Clinical Pharmacy Services Division, Pharmacy Services Department, Johns Hopkins Aramco Healthcare, Dhahran 31311, Saudi Arabia; 6 Department of Informatics and Outcomes, St Joseph Mercy Oakland, Pontiac, MI 48341, USA; anupam.a.sule@stjoeshealth.org; 7 Department of Medicine Keystone Health, Penn State University School of Medicine, Hershey, PA 16801, USA; tirupa@keystonehealth.org; 8 Department of Medicine, Wellspan Chambersburg and Waynesboro (Pa.) Hospitals, Chambersburg, PA 16801, USA; 9 Research Center, Almoosa Specialist Hospital, Alahsa 36342, Saudi Arabia; abbas.almutair@almoosahospital.com.sa; 10 College of Nursing, Prince Nora University, Riyadh 11564, Saudi Arabia; 11 School of Nursing, Wollongong University, Wollongong, NSW 2522, Australia; 12 Administration of Pharmaceutical Care, Al-Ahsa Health Cluster, Ministry of Health, Alahsa 31982, Saudi Arabia; saalhumaid@moh.gov.sa; 13 Department of Pathology, Faculty of Medicine, Al-Azhar University, Cairo 11884, Egypt; abdulkarim.hasan@azhar.edu.eg; 14 Prince Mishari Bin Saud Hospital in Baljurashi, Ministry of Health, Baljurash 22888, Saudi Arabia; 15 Department of Microbiology, Punjab Agricultural University, Ludhiana 141004, India; manish-cobsmb@pau.edu; 16 The Trafford Group of Colleges, Manchester WA14 5PQ, UK; 17 Department of Veterinary Microbiology and Immunology, College of Veterinary Sciences, Uttar Pradesh Pandit Deen Dayal Upadhyaya Pashu Chikitsa Vigyan Vishwavidyalaya Evam Go Anusandha Sansthan (DUVASU), Mathura 281001, India; ruchitiwari@duvasumathura.com; 18 Division of Surgery, ICAR-Indian Veterinary Research Institute, Izatnagar, Bareilly 243122, India; sharunkhan@ivri.res.in; 19 Department of Chemistry, Government College of Engineering, Keonjhar 758002, India; rkmohapatra@gcekjr.ac.in; 20 Department of Pharmacy, Faculty of Pharmacy, University of Dhaka, Dhaka 1000, Bangladesh; saikat-2018926336@pharmacy.du.ac.bd; 21 Department of Pharmacy, BGC Trust University Bangladesh, Chittagong 4381, Bangladesh; 22 School of Life Science and Food Engineering, Huaiyin Institute of Technology, Huaian 223003, China; bilaluaf@hyit.edu.cn; 23 Division of Pathology, ICAR-Indian Veterinary Research Institute, Izatnagar, Bareilly 243122, India; rajendra_singh5747@rediffmail.com; 24 Department of Mathematics and Statistics, Imam Mohammad Ibn Saud Islamic University, Riyadh 11432, Saudi Arabia; saalyami@imamu.edu.sa; 25 WHO Collaborating Centre on eHealth, UNSW Digital Health, School of Public Health and Community Medicine, Faculty of Medicine, UNSW Sydney, Sydney, NSW 2052, Australia

**Keywords:** COVID-19, SARS-CoV-2, cytokine storm, disease management, dysregulation of immune system, immunotherapy

## Abstract

The pathogenesis of coronavirus disease 2019 (COVID-19), caused by severe acute respiratory syndrome coronavirus 2 (SARS-CoV-2), is still not fully unraveled. Though preventive vaccines and treatment methods are out on the market, a specific cure for the disease has not been discovered. Recent investigations and research studies primarily focus on the immunopathology of the disease. A healthy immune system responds immediately after viral entry, causing immediate viral annihilation and recovery. However, an impaired immune system causes extensive systemic damage due to an unregulated immune response characterized by the hypersecretion of chemokines and cytokines. The elevated levels of cytokine or hypercytokinemia leads to acute respiratory distress syndrome (ARDS) along with multiple organ damage. Moreover, the immune response against SARS-CoV-2 has been linked with race, gender, and age; hence, this viral infection’s outcome differs among the patients. Many therapeutic strategies focusing on immunomodulation have been tested out to assuage the cytokine storm in patients with severe COVID-19. A thorough understanding of the diverse signaling pathways triggered by the SARS-CoV-2 virus is essential before contemplating relief measures. This present review explains the interrelationships of hyperinflammatory response or cytokine storm with organ damage and the disease severity. Furthermore, we have thrown light on the diverse mechanisms and risk factors that influence pathogenesis and the molecular pathways that lead to severe SARS-CoV-2 infection and multiple organ damage. Recognition of altered pathways of a dysregulated immune system can be a loophole to identify potential target markers. Identifying biomarkers in the dysregulated pathway can aid in better clinical management for patients with severe COVID-19 disease. A special focus has also been given to potent inhibitors of proinflammatory cytokines, immunomodulatory and immunotherapeutic options to ameliorate cytokine storm and inflammatory responses in patients affected with COVID-19.

## 1. Introduction

Severe acute respiratory syndrome coronavirus 2 (SARS-CoV-2) emerged as the cause of the greatest pandemic disease of this century, coronavirus disease 2019 (COVID-19), and as of 2 May 2021, over 152 million confirmed cases have been documented, with above 3.2 million deaths across the globe [1,2,3]. It has caused the loss of more than 20.5 million years of life globally [4]. Although the source of this virus became known to be bats, with pangolins also being implicated, the origin of this virus is a matter of great debate, and the intermediate host(s) is being investigated; SARS-CoV-2 has also been reported from few animals and wildlife species as well as posing zoonotic concerns [1,5,6,7,8]. The effects of the disease have been observed in every country with a high dissemination rate. Both symptomatic and asymptomatic SARS-CoV-2 infection have been reported; the symptomatic infection may lead to moderate and severe clinical pathology of COVID-19 with varying general mortality rates of 2 to 5%, while in the majority of the population, the viral infection has no depleting effects and thus recovery occurs [9,10]. Nevertheless, the effect of the disease is highly intensified in patients with co-morbid conditions such as hypertension, cardiovascular vascular diseases, and diabetes [11,12,13]. High efforts have been made at the global level to design and develop effective drugs, therapies and vaccines against SARS-CoV-2 [14,15,16,17]. However, a few vaccines are now available, and vaccination has started in many countries to counter COVID-19 [17]. Though some repurposed drugs, immunotherapies and other therapeutic regimens are being applied for emergency use, especially in critically ill patients, effective drugs and therapeutics are yet awaited for treating patients with COVID-19 [16,18]. Of note, SARS-CoV-2 variant strains have also emerged (UK variant B.1.1.7, South Africa B.1.351 variant, mink variant and others), which may pose higher public health concerns [6,19].

SARS-CoV-2 primarily attacks the lower respiratory tract by entering into the lung cells via binding to the ACE2 (angiotensin-converting enzyme) receptors and causes severe lung damage, leading to severe acute pneumonia. However, many studies also verified other clinical manifestations including neurological, gastrointestinal, and renal abnormalities associated with SARS-CoV-2 infection [9,20,21]. Infection of SARS-CoV-2 in the lower respiratory tract activates the critical immune cells of the innate immune response, such as macrophages and neutrophils, which release several chemokines and cytokines to activate components of the adaptive immune system such as B and T cells. However, in severe cases, disturbance in normal immunological response due to some unknown factors leads to the hyperactivation of immune cells. It generates an abrupt and elevated cytokine level known as cytokine storm or hypercytokinemia [9,22].

A severe systemic injury caused by dysregulated cytokine release leads to organ damage in these patients, causing poor disease prognosis. Many promising disease prognostic markers are identified by researchers that can serve in managing the disease. The patients affected with severe COVID-19 experience neurovegetative symptoms (tachycardia) for many weeks or months after their recovery, suggesting the imbalance of sympathetic–parasympathetic activity of the autonomic nervous system [23,24]. Careful scrutiny on the characteristics of the viral particles, disease progression, immune response, and clinical manifestations are significant in managing the disease. Hence, the present review focuses on several parameters, including the role of cytokine storm in generating acute respiratory syndrome, the variable outcome of the disease in a different race, gender, age, and the factors affecting the immunopathology of COVID-19 and immune response against the SARS-CoV-2 infection. Furthermore, the immunomodulatory strategies are also discussed to control the cytokine storm or hypercytokinemia. 

## 2. Immuno-Inflammatory Characteristics in Patients with COVID-19 Based on Symptoms

The clinical manifestations of COVID-19 disease are primarily reported to be respiratory distress, often coupled with high-grade pyrexia and pneumonia in severe cases. Inflammatory markers such as cytokines, interferons, and interleukins are potential culprits for serious COVID-19-related complications [25]. Earlier, the World Health Organization (WHO) categorized the clinical symptoms of COVID-19 primarily to respiratory manifestations such as coughing, throat soreness, and dyspnea [26]. However, a clear pathway for viral replication and pathology of the disease has not been clearly identified. Evidence-based research has indicated that some patients may experience gastrointestinal symptoms suggestive of COVID-19 viral replication in the digestive system [27,28]. For example, Duan et al. described the immune-inflammatory attributes in COVID-19-infected individuals associated with digestive manifestations by splitting patients into three categories [29]. The first and second patient groups showed only digestive and respiratory symptoms, respectively, while both the respiratory and digestive symptoms were displayed in the third group. In patients with COVID-19, commonly observed abnormalities were mild liver injury and triggering of the immuno-inflammatory system. In a recent study, 43.4% of critically ill patients with COVID-19 showed damage to liver functions to varying extents [30]. At the beginning of this pandemic, the clinical significance of liver involvement was debated. In this context, Nardo et al. have discussed the potential pathophysiological mechanisms and the possibility of long-term liver injury due to hyperinflammatory response in severely infected patients with COVID-19 [31]. As per the evidence-based studies, the elevated liver transaminases (AST and ALT) in patients with COVID-19 reflect hepatocellular damage and may be accompanied by bilirubin levels [32,33,34,35]. Additionally, some recent metanalyses highlighted a significant increase in cholestatic liver enzymes (ALP and γ-GT), which reflect cholangiocellular injury [32,33,34,35].

It is also worth noting that patients with no pre-existing medical problems still had minor signs of liver injury, while patients with a serious infection and elevated viral load had a substantial liver impairment and immune system destruction. Chai et al. observed higher expression of ACE2 receptors in bile duct cells and liver cells relative to alveolar epithelial type II cells, supporting these findings [36]. Given the importance of bile duct cells in immune defense and liver regeneration, their dysfunction may be a significant cause of virus-induced hepatic damage in COVID-19 patients. However, the interrelationships between liver injury and respiratory failure have yet to be resolved [37,38,39].

Patients associated with digestive manifestations showed significantly high levels of inflammatory cytokines [40]. Moreover, a meaningful correlation was observed between proinflammatory cytokines such as TNF and IL-2 concentration in the poor prognosis of the disease among the patients with pre-existing gut-related health conditions such as inflammatory bowel disease (IBD) [40]. In this context, a clinical study constituting of 204 patients suffering from COVID-19, 103 patients revealed gastrointestinal-specific symptoms, such as vomiting, diarrhea, lack of appetite, and abdominal disturbance [41]. In these cases, six patients showed digestive indications but lacked respiratory manifestations. The gastrointestinal symptoms were observed to increase the severity of the disease [41]. In a study involving 206 patients with mild COVID-19, 48, 89, and 69 patients presented digestive, respiratory, and both respiratory and digestive symptoms, respectively. Although patients with gastric symptoms were given care later than individuals with respiratory indications, patients with digestive symptoms presented an extended period between the onset of symptoms and viral eradication. In contrast to respiratory symptoms, patients with digestive signs showed a high HIV-positive ratio in stool samples [42].

Numerous reasons are speculated to describe why digestive symptoms occur in the case of COVID-19. First, SARS-CoV-2 binds to the ACE-2 receptor for invading the human body to induce hepatic tissue damage by upregulating the expression of ACE-2 in liver tissues [43]. Secondly, it directly or indirectly harms the gastrointestinal system via inflammatory responses. Reports have shown the detection of viral nucleic acid in fecal specimens up to 53.4% of patients with COVID-19 [44,45,46]. Enteropathic viruses result in digestive symptoms by directly damaging the intestinal epithelia; however, additional investigations are required to substantiate this speculation. Third, the viral particles might impact the gut microbiota, which triggering the gastric manifestations. The diverse and astonishing numbers of intestinal flora play a number of pivotal roles in the human body, such as nutrition metabolism, regulation of maturation and development of the immune system, and antimicrobial properties [47]. Lastly, variations in the functions and composition of the digestive tract flora can significantly impact the prognosis of the disease [48,49].

Several reports also suggested neurological manifestations related to SARS-CoV-2 infection [50,51,52,53,54,55,56,57,58]. The presence of SARS-CoV-2-RNA has been identified in cerebrospinal fluid in only a few cases and even in an autopsy brain specimen in one case [50]. The presence of SARS-CoV-2 receptors in the nervous system can be linked with neurological manifestations related to COVID-19, such as stroke, polyneuropathy, acute encephalitis, and brain inflammation; the authors of [51,52,53] reported the neurological attributes by analyzing the CSF of 30 COVID-19 individuals. The most frequently observed symptoms were impaired consciousness, anosmia, altered mental status, ageusia/hypogeusia, new paresis, seizures, and hypo-/areflexia [52,58]. Common neurological diagnoses were likely to cerebrovascular events, (poly) neuropathy, and encephalopathy. These outcomes are in accordance with earlier studies of encephalopathies, cerebrovascular events [52,54], and autoimmune neuropathies [55] in patients with COVID-19. Endotheliitis might explain cerebrovascular events during SARS-CoV-2 infection [56], whereas autoimmune neuropathies also indicate indirect involvement of the CNS (central nervous system). Furthermore, the hyperactivated inflammatory response is also linked with the increased risk of neurological comorbidities with COVID-19. The excessive release of inflammatory factors disturb the coagulation system, which may enhance the D-dimer concentration aggregation of platelets [57,58]. Thus, the characteristics of inflammatory markers are distinct based on the clinical manifestations and play variable roles among different clinical conditions. He et al. have explored the relationship between D-dimer levels, clinical classification and prognosis of clinical outcomes of 1114 patients with confirmed COVID-19 [59]. As per the study, D-dimer levels are abnormal in severe and critical patients as compared with mild patients. Moreover, significantly higher D-dimer levels were observed in patients who had died as compared with surviving patients. Hence, D-dimer may be used to evaluate the prognosis of patients affected with COVID-19, and also, the patients with male gender, greater age, dyspnea, etc., have a higher D-dimer value (*p* < 0.05).

## 3. Correlation between Interleukins and TNF Levels in COVID-19 Related to Respiratory and Digestive Symptoms

An unregulated cytokine storm greatly steers the fatality of COVID-19. Cytokine storm is a remarkable pathologic condition influenced by amplified production of interleukins, especially IL-6 (interleukin-6). Production of IL-6 and activation of transcription 3 (STAT 3) indicates activation of the nuclear factor kappa B (κB) pathway leading to ARDS-specific symptoms [60]. It has been established by various research reports that patients of COVID-19 with poor prognostic traits suffer from diverse medical implications such as multiple organ breakdown and thrombosis [61]. In the event of severe lung infection due to COVID-19, hyper-production of inflammatory markers such as IFN-γ, TNFα, IL-1, IL-6, and IL-12 are evident. The viral particles enter the cell through the ACE2 receptors with the help of endosome-specific receptors such as TLR-7 [62,63]. This activation triggers the formation of inflammatory markers such as TNF-α and interleukins 2 and 6, which in turn generates the production of CD8^+^ T cells. In response to this hyperinflammatory reaction, thrombosis occurs in the small vessels of the lungs. It leads to serious complications in alveoli such as disturbance in gaseous exchange and leaking of several other factors into the lungs. In some severe cases, hyperinflammatory response causes disseminated intravascular coagulation (DIC), leading to lung failure [64,65]. 

In the digestive system, viral entry and replication occur primarily through the attachment of the viral particles to the ACE2 receptor. Since the ACE-2 receptor is expressed in high levels in the cholangiocytes, derangement of liver function can be relatively challenging [36]. The viral dissemination in the digestive system is rather subtle compared to the respiratory infection and cannot be detected in the regular rRT-PCR (real time-PCR) test. Thus, patients with gastrointestinal infection who were declared negative in the rRT-PCR test may experience serious gastro-intestinal complications if untreated. The presence of RNA nucleic acid in fecal specimens after viral clearance in the lungs indicates the complicated pathogenesis of the virus. Disease severity in COVID-19 has been always associated with acute respiratory disease syndrome (ARDS), and primary markers for gastrointestinal (GI) infection have not been well-established. Duan et al. studied the inflammatory markers in patients who exhibited gastrointestinal manifestations due to COVID-19 [29]. They reported that mild hepatic damage was the common phenomenon observed in patients who had gastrointestinal infection. The titers of inflammatory cytokines such as IL-2, 4, and 10 were slightly higher than in patients with ARDS. Several other studies also linked abnormal immune functioning, such as elevated levels of cytokines, i.e., cytokine storm or hypercytokinemia, lymphopenia, and reduction in the proliferation of CD4^+^ T cells during SARS-CoV-2 infection with the hepatic injuries and severe liver damage in some cases [46,66,67,68].

However, liver injury generally corresponds to an increased concentration of liver parameters such as SGOT (serum glutamic oxaloacetic transaminase) and SGPT (serum glutamic pyruvic transaminase) [29]. Extreme liver damage may be due to factors such as direct dissemination of the viral particles in the liver, hepatic injury due to immune aggression or drug-driven toxicity [69]. In a meta-analysis of 23 observational studies, the three factors, namely: concentration of inflammatory markers, titer values of immune proteins CRP (C reactive protein), TNFs (tumor necrosis factors), interleukins (especially IL-6), and disease severity, were proportional to each other. The predominant marker for liver damage due to COVID-19 is the C-reactive protein. The secretion of IL-6 and T cells regulates the production of this pentameric protein. A high concentration of CRP in plasma and systemic circulation can indicate hepatic damage. Excessive production of inflammatory elements can induce the production of CRPs, leading to what is called the cytokine storm.

## 4. Factors Influencing Inflammatory Cytokine Production by Modulation of the Immune System

The severity of COVID-19 is often directly proportional to the concentration of inflammatory markers and other specific immune proteins. Reports have signified that COVID-19 disease with GI manifestations is generally more severe (23%) compared with COVID-19 disease without any GI manifestations (8%). These reports signify that the viral infection in the GI tract may aggravate the production of mucosal cytokines and thereby affect the disease prognosis. Xiao et al. reported findings of innumerable infiltrated plasma cells and lymphocytes in the lamina propria of gastrointestinal organs, indicating viral infection with interstitial edema [46]. Additionally, sodium levels in the blood become dwindled due to electrolyte instability [26].

Interferon, especially IFN-γ expressed during inflammatory bowel disease (IBD), triggers ACE2 expression through cytokine signaling events [40]. Furthermore, the available data also suggest the role played by IFN-λs in the disease pathology. IFN-λs may contribute to the disease severity in patients infected with SARS-CoV-2 by exacerbating innate immune responses, especially in the case of chronic or severe disease states [70]. In some patients, following the primary attack in the alveolar lung cells, a secondary viraemia is evident chiefly targeting the intestine and the renal system [71]. Studies reveal the presence of SARS-CoV-2 RNA in stool samples of patients with COVID-19, suggesting viral dissemination up to gastrointestinal tracts [46,72]. Many factors influence the high disease lethality in patients with COVID-19, especially the elderly. Qin et al. have indicated that the lower concentration of lymphocytes, monocytes, eosinophil, and basophil count, and high neutrophil titers are associated with disease severity [13].

Besides the cytokines, several other small chemical molecules or chemokines such as CCL2 (MCP1), CCL3 (MIP1α), CXCL10 (IP-10) have been reported to be higher in the serum of severely ill patients with COVID-19 [73,74,75,76]. These chemokines may correlate with the increased level of cytokines as they act as chemoattractants for several immune cells and lead to the cytokine storm via modulating the immune response in severe cases [75,76,77,78,79]. For example, Chi et al. detected the production of pro-inflammatory chemokines and cytokines in the serum samples of asymptomatic as well as symptomatic patients [80]. The presence of these chemical molecules, including IL-6, IL-18, IL-10, IL-7, M-CSF, MIG, G-CSF, MCP-3, IP-10, MCP-1, and MIP-1α, were related to the disease severity. In addition, chemokine and cytokine profiles were substantially greater in COVID-19 infected males than female counterparts, as reported in some other reports [30,81]. The concentration of G-CSF, MCP-1, and VEGF in serum was found to be positively associated with viral loads. Zhao et al. [82] reported a significantly increased chemokine level in the initial phase of COVID-19 infection. The early generation of IL-1RA and IL-10 were found to be correlated with the severity of the disease. The level of most cytokines, including IFN-γ and IL-6, was increased in the later phases of the disease, whereas TNF-α and GM-CSF presented no meaningful differences between mild and severe cases [76,77,83]. Furthermore, NLRP3 inflammasome releases proinflammatory cytokines (IL-1β, IL-18) and triggers gasdermin D-mediated pyroptotic cell death, as a result of which elevated level of enzyme lactate dehydrogenase (LDH) is observed in the blood of patients with COVID-19 and correlated with disease severity [78,84,85,86].

## 5. Immunomodulatory and Immunotherapeutic Approaches to Counter Cytokine Storm

Cytokine storm is a major immunological problem that leads to disease severity. The condition in itself is more lethal than the viral infection, and no single treatment modality has proved to be effective. Evidence-based treatment regimens and identifying the choice of drugs for COVID-19 disease are being explored. Presently, the treatment strategies are supportive types with few drugs and therapies being administered to reduce the severity of SARS-CoV-2 infection in patients [87,88,89]. In every country, the treatment strategies vary depending on local scientific anecdotes or interim results of local studies. Many drugs that are administered to patients with COVID-19 were agents used to treat earlier viral epidemics such as severe acute respiratory syndrome (SARS) and the Middle East respiratory syndrome (MERS) [90,91]. The cases of COVID-19 should be directed to control and suppress the activity of pro-inflammatory cytokines, thereby preventing cytokine storm/hyperinflammatory stage and ameliorate the severity of the disease in affected patients. This can be achieved with the help of immunomodulator and immunosuppressor drugs [92]. Several immunomodulatory and immunotherapeutic options are being evaluated to control the elevated cytokines levels in severely infected patients with COVID-19, such as antiviral drugs, corticosteroids, interleukins antagonists, monoclonal antibodies, mesenchymal stem cells, herbal medicines and others [16,89,91,93]. Convalescent plasma, antibodies and intravenous immunoglobulins are potent immunomodulatory therapeutics being used to treat severely ill patients with COVID-19, modulate and/or block cytokine storm and help in reducing mortality [16,18,94,95,96,97]. Mesenchymal stem cells (MSCs), owing to their strong immunomodulatory and regenerative properties, constitute promising avenues in treating and managing patients with COVID-19, decreasing the severity and mortality during SARS-CoV-2 infection, ameliorating ARDS and controlling cytokine storm, as observed in recent clinical trials; these therefore need to be explored to their full potential to counter the COVID-19 pandemic and other challenges in the future [98,99,100,101,102,103].

Targeting crucially involved cytokines such as IL-6, IL-1β, IL-12, IL-1, TNF-α, and inhibiting cytokine storm-inducing signaling pathways such as JAK, JAK/STAT, and NF-κB have been involved as effective strategies to modulate the hyperinflammatory response against SARS-CoV-2 infection [79,104]. Hence, combinations of anti-inflammatory drugs such as JAK inhibitors, IL-1 inhibitors, IL-6 inhibitors, colchicine, TNF-α inhibitors are used to manage uncontrolled immune dysregulation [105,106]. In a typical human body, the expression of IL-6 is highly regulated and can stimulate the expression of only selected immune modules such macrophages, neutrophils, and T cells. However, during severe viral infection owing to COVID-19, this regulation is highly disrupted, leading to uncontrolled wide-range signaling leading to ‘cytokine release syndrome’. In this reference, many anti-IL-6 antibodies such as sarilumab, tocilizumab, and siltuximab have been explored by many researchers [69,106,107]. These antibodies can stifle the key pathways that lead to immune dysregulation causing early cell senescence and oxidative stress, thereby leading to improved clinical outcomes and better disease prognosis. Animal and clinical studies on SARS-CoV-2 have revealed that blockage of the transcription factor kappa-B (NF-κB) can reduce IL-6 expression, and can be a potential target to treat critically ill patients with COVID-19 [108,109]. Therefore, the experimental animals were injected with viral particles devoid of the viral envelope protein that can express the transcription factor NF-κB. This study showed increased survival rates and low IL-6 concentration [110]. Many studies on the safety and efficacy of antibodies and monoclonal antibody-based therapies in treating COVID-19-induced immuno-dysregulation are presently conducted worldwide [16,18]. Consequently, among a range of the above-mentioned monoclonal antibodies, tocilizumab (TCZ) has been reported as a significantly better therapeutic candidate to treat the cytokine release syndrome and approved for the treatment of critically ill patients with SARS-CoV-2 infection [111]. Moreover, fever and the acute inflammatory response are mediated by pro-inflammatory cytokines, including IL-6 (a critical cytokine), C-reactive protein and ferritin, and TCZ to lower the fever and IL-6 levels, which in turn reduce the hyperinflammatory response. Furthermore, TCZ has been used to treat COVID-19 in various chronically ill hospitalized patients in several clinical trials. It is also used in those with severe COVID-19 symptoms and those with advanced illness. In this context, an uncontrolled clinical study in China released the results of patients who were treated with TCZ. In this uncontrolled study, tocilizumab at 400 mg was administered subcutaneously to the patients with moderate COVID-19 pneumonia, and TCZ has been reported as effective and reliable in treating hospitalized patients [112]. Furthermore, baricitinib, a potent inhibitor of the JAK-STAT receptor, was found to suppress the invasion of SARS-CoV-2 into the host cells. This drug can also control the hyperinflammatory phase and can be considered a therapeutic option [111,113,114]. The inhibitory activity on the JAK pathway is mediated by the suppression of signal transduction initiated with the binding to the IL-6 receptor. Therefore, the use of immunosuppressive drugs that inhibit JAK/STAT pathway can be considered as a therapeutic strategy to manage the hyperinflammatory response [113,115,116]. Similarly, ruxolitinib is another JAK-STAT antagonist that can inhibits IFN-γ [117,118]. TNF-α inhibitors such as adalimumab and golimumab can also be employed to manage COVID-19-induced hyperinflammatory response [119]. Furthermore, suppressing TNF-α induces a decrease in IL-1 and IL-6, along with adhesion molecules and vascular endothelial growth factors (VEGFs), and TNF blockers are being recommended for use in hospitalized patients with absurd inflammatory reactions in disorders such as autoimmune diseases [120]. Although findings from the clinical trials do not support the widespread use of IL-6 antagonists/inhibitors in hospitalized patients with mild-to-moderate COVID-19, the use of IL-6 antagonists/inhibitors can produce a beneficial outcome when utilized in patients with severe disease [121,122]. Moreover, clinical experiments should be conducted in the future to discuss these associations that can be harnessed in developing effective therapeutic regimens against COVID-19. 

In various research studies, a plethora of corticosteroids, including dexamethasone, methylprednisolone, and prednisone have been explored to modulate the hyperinflammatory response in severely ill patients with COVID-19 [123]. Among several corticosteroids, dexamethasone has been reported as a potential anti-inflammatory agent to modulate the immune response to control elevated levels of cytokines in severe cases. However, the use of dexamethasone has only been found as a respiratory support in COVID-19. Many earlier reports have connected the identification of inflammatory markers with the progression and severity of COVID-19 [57,61,78,124,125]. In agreement with these studies, the utilization of dexamethasone is useful only in patients needing respiratory provision, and individuals not necessitating respiratory care were unlikely to benefit from dexamethasone [126,127]. It has been revealed that the consumption of corticosteroids impairs virus eradication in SARS-CoV-1- or SARS-MERS-infected individuals [128,129]. Additional studies assessing viral titers and inflammatory markers using dexamethasone are required to elucidate these results in COVID-19 [83].

The complement system inhibitors, including eculizumab, ravulizumab, tesidolumab, and vilobelimab (IFX-1), may be considered as essential therapeutic drug candidates to control the unregulated release of inflammatory cytokines or inflammatory response [130,131,132,133]. Among the range of complement system inhibitors mentioned above, tesidolumab, a C5-blocking monoclonal antibody that prevents the formation of C5a and the membrane attack complex, was found to be effective in reducing the exaggerated inflammatory reaction and promoting clinical improvement in patients suffering from COVID-19 with severe disease [132]. Clinical studies, including the use of C3 blocking peptides such as AMY-101, are in the progress of illustrating a potential regulation in the aggravated inflammatory reaction, lung damage, and multiple organ failure, which have been found in some COVID-19 instances. AMY-101, a cyclic peptide C3 blocker, is being used as an experimental drug in patients with COVID-19 with severe organ failure. The hyper-inflammatory response was shown to be significantly reduced 48 h after therapy began [130]. Moreover, the inhibition and regulation of the complement system through various drug candidates can provide a more comprehensive therapeutic approach against complement-mediated hyper-inflammatory response. 

In addition, traditional Chinese medicine (TCM), a branch of the medical system that can offer several concoctions/preparations containing rare herbal ingredients, has provided a few preparations possessing significant bioactivity that can be used for treating COVID-19 [134,135]. The major biological activities exhibited by TCM preparations such as anti-inflammatory and immunomodulatory activity, can be utilized for treating patients with COVID-19 [135]. Monotherapy using TCM is not advised as they are mostly beneficial in the role of supportive agents. However, they can be used in combination with Western medicines. The combined use of Western medicines and TCM in patients with COVID-19 was found to reduce the duration of hospital stay due to the faster rate of recovery [136]. Lianhuaqingwen, a TCM preparation with broad-spectrum antiviral activity, was previously found to exert immune regulatory activity when used against respiratory viruses [137]. Recently, Lianhuaqingwen was found to inhibit the replication of SARS-CoV-2 during in vitro studies. It also suppressed the production of pro-inflammatory cytokines such as IL-6, TNF-α, monocyte chemoattractant protein 1 suggesting potent anti-inflammatory activity [134]. Similarly, another study has evaluated the potential of a TCM formula, Babaodan, for its ability to suppress cytokine storm. The anti-inflammatory activity of Babaodan was assessed in vivo using a mouse model (post-viral bacterial infection-induced pneumonia model). The findings from the in vivo study confirmed the potential of Babaodan to inhibited the release of IL-6. The protective role is believed to be mediated by inhibiting the NF-κB and MAPK signaling pathways [138].

Apart from that, a variety of phytoconstituents present in clinically significant plants appear to have proven immunoregulatory potential in monitoring and maintaining hyper-inflammatory responses and are thus being researched for prospective treatment and prevention efficiency in patients with COVID-19 [7,139,140,141,142,143]. Novel anti-TNF and anti-IL-6 extracts from *Cannabis sativa* have recently been demonstrated to be useful in regulating the uncontrolled production of inflammatory cytokines [144]. In animal cell lines, *C. sativa* has been verified to reduce the expression levels of IL-6, IL-8, C-C motif chemokine ligands (CCLs) 2 and 7, and ACE2, suggesting that it may be a valuable contributor to existing anti-inflammatory regimens for the management of COVID-19 [145]. Moreover, multiple micronutrients, including vitamin D and zinc, have been identified as significant determinants for the intensity of inflammatory response in viral infections such as SARS-CoV-2. In addition, vitamin D deficiency has been linked to respiratory diseases, with deficiency leading to acute respiratory illnesses [146]. Several studies have identified the importance of vitamin D in controlling SARS-CoV-2 infection, and it has been proposed that vitamin D supplementation could be helpful for vulnerable individuals, such as elderly patients with chronic diseases and obese patients, by reducing the intensity of inflammatory response [147].

Therapeutics that selectively target the lungs have tremendous potential in managing COVID-19 as SARS-CoV-2 invades via lung ACE2 receptors causing severe pneumonia. Recent reports have confirmed the association between 25-hydroxycholesterol and cytokine levels [148,149,150]. The activation of inflammatory SREBP2- and NF-κB-mediated inflammasome signaling pathways play a major role in COVID-19 pathogenesis. Therefore, 25-hydroxycholesterol and di-dodecyl dimethylammonium bromide nanovesicles can be considered as potential drug candidates that restore the intracellular cholesterol level, thereby inhibiting cytokine storm [150,151]. The findings indicate that 25-hydroxycholesterol and di-dodecyl dimethylammonium can downregulate NF-κB and SREBP2 signaling pathways. Furthermore, they can selectively accumulate in the lung tissues, reducing inflammatory cytokine levels [150]. Other than standard therapeutic options, numerous molecular intervention approaches are being studied to tackle the cytokine storm, such as targeting the microRNAs that are required for cytokine development. MicroRNAs and microRNA network targeting may be a promising development for designing quicker and more efficient therapeutic methods for SARS-CoV-2 infection-induced hyperinflammatory response [152]. Several therapeutic candidates with various chemical natures and mechanisms of action are being investigated and reported to regulate the uncontrolled and hyperinflammatory response. Tocilizumab, sarilumab, ruxolitinib, anakinra, and baricitinib, combined with dexamethasone, can be considered extremely effective and reliable therapies for controlling an uncontrolled immune response and hyperinflammatory response [153,154,155].

## 6. Degree of Difference in Cytokines Storm in Relation to Age, Sex, and Co-Morbidities

Research indicates that COVID-19 disease susceptibility is widely assorted in terms of age, sex, and immunity status. It has been found that the male population is more susceptible to infections compared to females due to several factors such as hormones, sex chromosomes, and general immunity. The two X chromosomes in females also regulate the expression of genes for the immune system leading to a higher percentile of innate immunity than males. Additionally, the presence of two X chromosomes in the female population leads to the overexpression of immune modules such as FOXP3, TLR-7, TLR-8, CD40L, and CXCR3 during an exigency such as viral infection [156]. The presence of this two-fold immunity steers more resistance against viral infections and evidently low viral load and less inflammation. Thus, women are certainly more immune to diverse diseases such as tuberculosis, malaria, AIDS, measles, mumps, influenza, and other infectious maladies [157]. Evidence-based research reveals that women have a high titer of immunoglobulin in circulation compared to males. Many reports worldwide testify that male patients (approximately 25%) are more susceptible to lung infections than females. In this regard, sex hormones can regulate the immune reaction during infections, and the higher levels of testosterone, a well-known male hormone, have been linked with lesser antibody production [158]. However, there is still no concrete evidence to prove the immunological differences in male and female patients with SARS-CoV-2 infection. Several analyses also inked the higher susceptibility of the male patients with the behavioral factors [159].

According to the most recent data, male patients are more likely to exhibit increased mortality rates than their female counterparts with similar age and ethnic backgrounds, indicating a clear sex-specific bias in SARS-CoV-2 infection [116,160,161]. Some countries have also reported that males are more likely to be exposed to SARS-CoV-2 than females [162]. Similarly, COVID-19 in male patients are also associated with more serious manifestations and a subsequent higher fatality rate even though some variations have been reported in certain countries [163,164]. Several countries such as Greece, Dominican Republic, Romania, and the Netherlands have reported high male/female ratios in terms of COVID-19-associated mortalities [162,164,165]. TMPRSS2 is a serine protease that contributes to the process of receptor mediated entry of SARS-CoV-2 by priming the Spike glycoprotein [166]. Single-cell RNA sequence analysis has confirmed that the expression of TMPRSS2 is higher in the case of males than females [167]. However, the relationship between the expression of TMPRSS2 and lung immunity remains poorly understood. Therefore, considering the role played by sex-specific bias in deciding the outcome of COVID-19, further studies are required to develop specific therapeutics that can overcome this shortcoming [167].

The sex-specific association was also reported, linking vitamin D status of the patients and the COVID-19 mortality rate, especially in the case of the older population [168]. The vitamin D status of the patient might play an important role in deciding the COVID-19 mortality rate due to the role played in immune system regulation as well as on the renin-angiotensin system [168,169]. Therefore, vitamin D might predispose a susceptible individual to SARS-CoV-2 infections [170]. Based on a study conducted in Switzerland, older males are highly susceptible to vitamin D deficiency compared to females [171]. Furthermore, males are more susceptible to ACE2 receptor dysregulation since vitamin D deficiency can be associated with increased X-chromosome-linked RAS activity [169]. Therapeutic supplementation of vitamin D might help to control the COVID-19-associated hyperinflammatory stage/cytokine storm by limiting the rapid increase of circulating pro-inflammatory cytokines [171]. 

The disease severity is related with age (over 65 years) and gender (men are more susceptible than women), while young children are less severely affected. Moreover, post-infectious hyperinflammatory disease is another outcome after SARS-CoV-2 infection. Brodin has discussed the immune-system differences between old and young people and men and women and other associated immunological factors towards the disease presentations and severity [172]. As per the previous studies, a disturbance in fertility in male patients is also expected due to the hormone-regulated expression of the ACE2 receptor [173]. However, the relationship between SARS-CoV-2 and renin-angiotensin system (RAS) in male reproductive apparatus is still unclear. Age alone is an important bifurcation criterion in COVID-19 susceptibility [174,175]. The geriatric populations succumb to other risk factors such as hyperlipidemia, hyperglycemia, cardiovascular diseases, and lung diseases [176]. The lethal combination of age-related immune dysregulation and co-morbid conditions causes the unregulated release of cytokines. This condition leads to intravascular coagulation in different organs causing hepatic injury, kidney damage, cardiovascular damage, and even death [177,178]. However, the relationship between aging and COVID-19 disease pathogenesis is not clearly known. Some factors such as glycation, inflammasome-induced pyroptosis, and aging are interrelated. Both innate immunosenescence and adaptive immunosenescence are exhibited as age advances causing incompetent pathogen identification, reduced natural killer cells, thymic atrophy, and inflation of languid memory lymphocytes [179]. It has also been reported that pediatric patients are recovered with a good survival rate compared to the adult population [180]. 

The clinical manifestations of COVID-19 are complex, ranging from mild fever to critical organ damage depending upon the immune capability of each individual. The acute respiratory symptom is the common clinical manifestation in patients suffering from severe COVID-19 with life-threatening effects such as metabolic acidosis, acute respiratory failure, septic shock, and coagulopathy. Only a few reports have specified gender-based differences in clinical characteristics and outcomes. Jin et al. reported that male patients had a high concentration of serum creatinine, white blood cells, and neutrophils [26]. Additionally, the mortality rate in the male population (70.3%) was higher compared to female patients (29.7%), although the disease susceptibility is distributed equally across genders. Some researchers compare this trend to the demographic data of longer life expectancy in women than men in some countries such as China. Moreover, similar trends were reported in European countries including Switzerland, France, and Germany [181,182]. In these countries, the male ratio of hospitalizations was three- to four-fold higher than women [183]. Both SARS-CoV-2 and SARS-CoV viral particles invade human cells through the ACE-2 receptor of target organs, indicated by specific organ damage [184]. Some researchers have also specified that the high expression of ACE-2 protein is associated with organ damage [185]. Reports reflect that levels of protein-specific ACE2 receptors are more abundant in males than in females [185,186]. ACE2 protein is involved in regulating cardiac function during a cardiac crisis such as myocardial infarction. Hence, high expression of ACE-2 protein can act as a prognostic marker in COVID-19. Tissue specimens from cadavers of SARS patients who succumbed to myocardial infarction showed low expression of ACE-2 protein and high concentration of viral titers [187]. Aggregate data containing information of more than two lakh patients with COVID-19 and 14,000 deaths indicate that the male fatality ratio is relatively high compared to the female population among all age groups, particularly middle-aged groups [188]. Furthermore, Parpia et al. have studied the racial disparities among 6065 COVID-19-related deaths in Michigan, USA, and found that in all strata, Black individuals are at higher risk of mortality than White individuals [189].

## 7. Key Elements of Innate and Adaptive Immune Responses

Regulation of the immune system is a primary prognostic criterion in patients with COVID-19. Any functional immune system will identify the pathogenic entry, apprise the system, annihilate the viral dissemination and clear up the clutter of dead particles. During COVID-19, modules of all three lines of immunity viz.—innate immunity, adaptive immunity, and passive immunity—are activated. Soon after the viral entry, antibody production is triggered by B and T cells. A plethora of antibodies with different dynamics and responses against SARS-CoV-2 has been investigated and among a pool of antigen-specific antibodies including IgA, IgM, IgG; IgA antibody-mediated immune response has been found as a stronger and more profound response against SARS-CoV-2 infection [190]. The T-lymphocytes also incite cellular immunity. T-helper cells are involved in the adaptive immune response, such as in cytokine release [191]. The release of cytokine CXCL10 at an early stage is a primary predictive marker of the disease course. A fully developed antigen-specific antibody is capable of neutralizing viral particles. 

The immune modules of the innate immune system express different pathogen-recognition receptors (PRRs) that study the molecular pattern of the invading pathogen [192]. These recognition patterns include C-type lectin receptors, NOD-receptors, RIG-I-like receptors, and toll-like receptors (TLRs) [193]. The SARS-CoV-2 viral antigens are recognized by RNA-sensors, including the RIG-I receptors and the toll-like receptors TLR 2, 3, and 7. These receptors activate the β-cell nuclear factor kappa-light-chain-enhancer (NF-κB) and interferon regulatory factor 3 (IRF3). These factors are the primers that express the production of pro-inflammatory cytokines, chemokines, and IFN-1.

As age advances, all the four primary functions are deranged [194]. In the COVID-19 condition, it is not yet clear which specific functions are dysfunctional. Patients with severe infection are reported to have developed hyperinflammation due to uncontrolled titers of neutrophils, cytokines (specifically IL-6 and TNF-α), and decreased counts of monocytes, basophils, and eosinophils [13,195].

Macrophages have been found as key players in developing proinflammatory and innate immune response and are considered important immune cells in the pathogenesis of COVID-19. Moreover, neutrophils numbers have been found to be significantly higher in severe cases of COVID-19 compared to mild and low levels of infection. Furthermore, the impaired blood vessels in the lungs are characterized by a higher number of monocytes and lymphocytes in critically ill patients with COVID-19. Moreover, the lymphocytes concentration was reported to be significantly lower than the neutrophils, and it was linked with the severity of the prognosis of COVID-19 [190,196]. On the other hand, the natural killer (NK) cells number was recorded as significantly lower than the macrophages in severe cases [68]. 

Moreover, the involvement of complement system in the poor prognosis of the disease is yet to be explained as several scientists believe that the complement system may be protective against SARS-CoV-2 infection [197]. A complement system, for instance, can suppress SARS-CoV-2 infection in asymptomatic or moderate cases; however, due to its significant pro-inflammatory activity, it can also exacerbate local and systemic damage in some patients with severe COVID-19 [133].

In this context, a retrospective study constituting 57 hospitalized patients with COVID-19 reported nonsignificant differences in C3 and C4 serum levels among the critically and moderately ill patients with SARS-CoV-2 infection [198]. However, in contrast to this, another recent study reported that the plasma C3a, C3c, and MAC levels in patients with COVID-19 were substantially higher than in stable controls, i.e., patients with moderate infection [199]. In comparison, C3a and MAC plasma levels were also higher in critically ill patients than moderately ill patients with SARS-CoV-2 infection. Both classes of patients with COVID-19 had identical plasma C3c levels [200]. Similarly, Holter et al. discovered elevated levels of C4d, C3bBbP, C3bc, C5a, and sC5b-9 in patients with COVID-19, indicating systemic activation of the complement system [201]. Furthermore, the presence of sC5b-9 was linked to ARDS and hyper-inflammatory response in patients with COVID-19 [201].

## 8. Response Rate of Activated T-Cell Types in COVID-19

T cells make up the first line of defense and are involved in eliminating the viral particles and shield against further infections. CD4+ and CD8+ T-cells are the primary T-cell subsets that are used as bioindicators and prognostic immune markers during acute viral infections. In patients who recovered from COVID-19, multi-targeted T-cell subsets are triggered against structural and non-structural regions of the virus. Peng et al. who studied the response of T cells against the SARS-CoV-2 virus, reported that T-cell responses were specific to spike protein, membrane antigens, and nucleoprotein antigens with a multi-specific pool of 23 reactive proteins [202]. These multi-specific proteins exhibit heterogeneous protection against assorted viral invasions, including mutation and variable antigen presentation. Mapping the overlapped peptides assist in recognizing the viral particles/regions that are targeted by T cells. Unlike SARS-CoV-1, the immune response against SARS-CoV-2 is widely assorted; the former triggers T cells specific only for the spike antigen [202]. Peng et al. employed the IFN-γ-ELISpot immunoassay and intracellular cytokine staining to identify COVID-19 antiviral effector responses [202]. These assays identified multifunctional CD4+ and CD8+ effector T cells. In patients with mild COVID-19, the proportions of SARS-CoV-2-specific CD8+ T cells were significantly higher compared to the critically ill patients with SARS-CoV-2 infection. These reports emphasize the protective role of CD8+ T cells in COVID-19 disease, but the kinetics of T-cell response is still unexplored. However, this implied the potential of non-spike proteins in the development of reliable and efficient vaccine against COVID-19.

In patients with severe COVID-19, T cells specific to the spike protein were found dominating the other T-subsets. This observation was also reported by other researchers [203]. More studies on the viral load during infections and the concentration of T-cell subsets are warranted to understand the nature of immune response towards different viral antigens. Studies on the strength, magnitude, and release timing of the T-cell subsets are critical information that defines the prognosis of the disease [204].

Several studies have suggested the significant reduction of immunologically important cells which provide adaptive immunity, such as T-cells in severe cases of COVID-19 [205,206]. Hence, T-cells in, for example, CD4^+^ and CD8^+^ cell populations have been found to be significantly declined compared to cells that provide innate immunity, such as macrophages and neutrophils in mild to severe cases of the SARS-CoV-2 infection [207]. However, the symptomatic recovered patients showed higher counts of CD8^+^ cells compared to critically ill patients [68]. Furthermore, unusual changes, i.e., lesser degranulation and lower levels of IL-2, IFNs, have been found in morphological and physiological attributes in CD8^+^ T-cells [68].

## 9. Inflammatory Cytokines and Multiple Organ Damage

Near links have been established between the severity of the COVID-19 infection and the immune response as the researchers also reported a strong correlation between disease development and immune responses during the SARS outbreak. Patients who had severe SARS infection had minimal T-cell immune modules [208]. Amelioration in the T-cell subset concentration was evident in patients who recovered from the disease, leaving the concentration of T cells as a predominant diagnostic marker for SARS. Similar trends were found in NK cells and cytokine concentration as well [209]. Since SARS and COVID-19 share similar characteristics in some aspects, the same can be expected in COVID-19 diagnosis [210].

Severe adverse reactions occur in patients with COVID-19 having a previous history of cardiac disease. Reports also specify that severe COVID-19 can even cause cardiac damage to patients with no history of cardiac failure due to excessive production of proinflammatory cytokines [78,211]. Increased production of cytokines leads to conditions such as myocarditis and pericarditis, leaving cytokine-inhibitor therapy highly promising [212]. Additionally, high titers of proinflammatory cytokines are associated with plaque bust, ischemia, and thrombosis.

COVID-19-induced lung injury is based on the activation of diverse pathways enabling multi-inflammatory reactions. The involvement of thrombin, a clotting factor in the inflammatory pathway through the proteinase-activated receptors-1 (PARs), leads to excessive cytokine-induced lung damage. The PAR-1 pathway antagonist factors can serve as an effective anti-inflammatory therapy option in patients with lung injury [213].

Recently, it has been demonstrated that SARS-CoV-2 can infect various cells of the renal system, such as tubular epithelial cells and podocytes. This may lead to the development of several renal abnormalities, including acute kidney injuries [214]. Various studies reported the association of COVID-19 with abnormal renal functioning during the prognosis of the COVID-19 disease. Furthermore, various renal abnormalities such as proteinuria, hematuria, and acute kidney failure have been reported in severely ill patients with COVID-19 [21,215]. 

Furthermore, headaches, fatigue, smell and taste disorders, and decreased consciousness are among the neurological effects that have been linked to COVID-19. Although SARS-CoV-2 has the potential to gain direct access to the nervous system, SARS-CoV-2 has only been detected in the cerebrospinal fluid in a few COVID-19 studies. There has been evidence of a connection between SARS-CoV-2 infection and neuropathogenicity. Furthermore, the SARS-CoV-2 virus has been shown to have the ability to affect progressive neurological diseases such as Alzheimer’s, Parkinson’s, and multiple sclerosis [216]. The SARS-CoV-2 infection has been attributed to neurological abnormalities in a recent study, and is consistent with a variety of pathogenic pathways, including post-infectious processes, septic-associated encephalopathies, coagulopathy, and endothelitis. There is no definitive evidence that SARS-CoV-2 is neuropathogenic [217]. Despite many inconsistencies, research has demonstrated a connection between SARS-CoV-2 infection and neurological disorders, including Alzheimer’s disease [218,219,220,221]. Furthermore, a postinfectious inflammatory disease known as a multisystem inflammatory syndrome in children (MIS-C) has emerged as a major COVID-19-related medical condition [222].

## 10. Dysregulated Immune Response as a Crucial Pathogenic Factor for Organ Disorder

Dysregulation in the immune system leads to severe complications in patients with COVID-19 [13,223]. Viral invasion and unregulated host immune response cause direct cytopathic effects leading to fatality in patients suffering from severe COVID-19 [224,225]. Severe COVID-19 disease is marked by a highly impaired immune system exhibiting lymphopenia, activated lymphocyte concentration or dysfunction, anomalies information and the expression of granulocytes and monocytes, increased cytokine concentration and antibodies [226]. Patients with lymphopenia show a remarkably low concentration of CD4+T, CD8+T, NK, β-cells, and lymphocyte ratio. Other reports have also indicated a marked reduction in the concentration of T-helper memory cells (CD3+CD4+CD45RO+) [227]. Thus, lymphopenia can serve as a prognostic marker for severe COVID-19 disease, although in rare cases, the condition is expressed in non-severe and pregnant patients [228].

Dendritic cells play a predominant role in producing immune modules of both innate and adaptive immune systems. A reduction in the function of dendritic cells leads to a substantial delay in T-cell response. Plasmacytoid dendritic cell (pDC) elicits the production of interferon, especially IFN-1 (interferon-1), against viral infection. Depletion of IFN-1 and NK cells may heighten viral invasion [229]. The release of excessive of chemokines and pro-inflammatory cytokines by immunity cells results in a cytokine storm, which is among the leading cause of death in corona infection [78,230]. Amongst the inflammatory molecules, mediating chemicals secreted by neutrophils and IMMs are likely to exhibit extremely hostile effects. TNF-α and IL-1β trigger the synthesis of hyaluronan synthase 2 (HAS2) in alveolar epithelium, fibroblasts, and CD31^+^endothelial cells and thus markedly augment water absorption, the formation of fluid jelly in lungs resulting in difficult breathing [231]. ROS and NO increase permeability to the endothelial cells and immune cells extravasation, causing alveolar epithelial industry, impaired gas exchange, and respiration syndrome [232].

The unregulated production of proinflammatory cytokines, chemokines, T-cell subsets, and regulatory T-cells cause a haywire reaction, ultimately leading to an internal disaster. This disaster causes extensive damage to the specific organ, leading to severe cytopathic effects and extensive tissue injury. In patients with severe lung damage, both type I and type II alveolar cells are infected. The excited macrophages trigger multi-immune cell proteins, including interferons, proinflammatory cytokines, monocyte chemo-attractant proteins, and tumor necrosis factors. Stimulation of T-lymphocytes incites complement activation (C5a/C5aR1), causing extensive organ injury [233,234,235].

**Figure 1:**
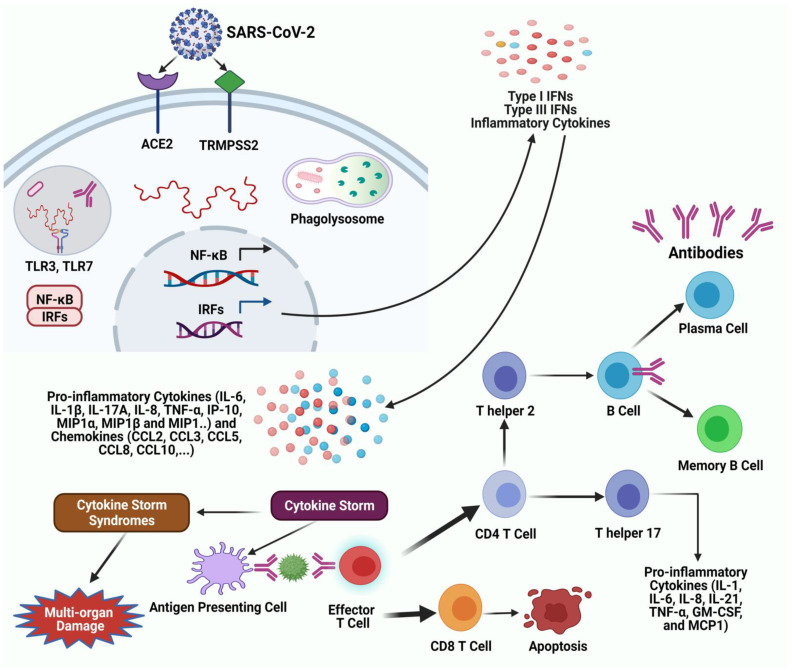
**Immune responses to SARS-CoV-2.** SARS-CoV-2 infects human cells in the angiotensin-converting enzyme 2 (ACE2 receptor) and the cellular serine protease TMPRSS2 for viral S protein priming. Endosomal and cytoplasmic sensors, toll-like receptor (TLR)-3 or -7 and mitochondrial antiviral-signaling protein (MAVS) are activated by viral RNA. Inflammatory cytokines and interferons (IFN) are contentiously induced by interferon regulatory factors (IRFs) and NF-κB, respectively. Most patients with severe COVID-19 exhibit considerably elevated serum levels of pro-inflammatory cytokines, including IL-6 and IL-1β, as well as IL-2, IL-8, IL-17, G-CSF, GM-CSF, IP10, MCP1, MIP1α (also known as CCL3), and TNF, characterized as a cytokine storm. The activation involved in the overproduction of cytokines (a phenomenon known as cytokine storm syndromes that cause respiratory distress) causes multi-organ damage. If an effector T-cell activates, it triggers the differentiation into CD4 and CD8 T cell. CD8 T-cell is capable of binding; it will undergo clonal expansion and immediately target infected cells by apoptosis through various pathways. If the CD4 T-cell is capable of binding, it can activate B-cells that recognize the antigen by causing them to clonally proliferate and secrete antibodies to target the SARS-CoV-2 virus. **Abbreviation:** ACE2, angiotensin-converting enzyme 2; COVID-19, coronavirus disease COVID- 19; TLRs, toll-like receptors; NF-κB, nuclear factor kappa-light-chain-enhancer of activated B cells; IRFs, interferon regulatory factors; IFN, interferons; IL, interleukin; TNF- α, tumor necrosis factor-alpha; MIP, macrophage inflammatory proteins; MCP-1, monocyte chemoattractant protein-1; IP, interferon gamma inducible protein; GM-CSF, granulocyte-macrophage colony-stimulating factor.

An overview of the generation of immune responses to SARS-CoV-2 is depicted in Figure 1.

## 11. Conclusions and Future Perspectives

SARS-CoV-2 infection is a complex phenomenon involving multiple immune factors and pathways. The virus initially dodges the scrutiny of host immunity, thereby increasing in number. The whacking viral concentration in the host cell causes severe disruption in the pathways that incite an immune response. Dysregulated immune proteins pile up, leading to signal collapse, causing the extensive release of immune modules leading to cytokine storm. The severity of SARS-CoV-2 infection has been linked with the hyperinflammatory response or cytokine storm that constitutes the uncontrolled and dysregulated secretion of inflammatory and pro-inflammatory cytokines. However, the prognosis of COVID-19 has been linked with the intensity of cytokine storm, and it may vary with age, gender, and race. Moreover, an effective immune response is critical to clear the viral infections, and any dysregulation in the immune response against SARS-CoV-2 infection leads to the severity of the disease. Various factors have been explored, such as significant T-cell reduction compared to macrophages and neutrophils, which have been linked to the dysregulation of immune response and cytokine storm. In addition, the unregulated activation of the complement system and the defect in IFN-I-mediated immune response might be considered as essential factors in the generation of a cytokine storm, which in turn leads to poor prognosis of the COVID-19 disease. However, due to the involvement of several factors in response to SARS-CoV-2 infection, many different antiviral therapies and immunomodulatory strategies such as the use of monoclonal antibodies (MABs), convalescent plasma therapy (CPT), corticosteroids along with complement system inhibitors that were successful in treating previous viral infections are explored in various ways to treat COVID-19. Many therapeutic approaches, such as CPT, along with multiple drug candidates, notably, tocilizumab, baricitinib, and dexamethasone, have shown significant potential in treating COVID-19 disease. However, in the future, an extensive understanding of the pathogenesis of the SARS-CoV-2 infection and molecular dissection of immune response generating pathways is needed to study presently available treatment options as well as to develop novel and effective therapeutic drug candidates. 

## Data Availability

Available data are presented in the manuscript.
